# Robust Interval Prediction of Intermittent Demand for Spare Parts Based on Tensor Optimization

**DOI:** 10.3390/s23167182

**Published:** 2023-08-15

**Authors:** Kairong Hong, Yingying Ren, Fengyuan Li, Wentao Mao, Xiang Gao

**Affiliations:** 1 China Railway Tunnel Group, Zhengzhou 450001, China; 2 School of Computer and Information Engineering, Henan Normal University, Xinxiang 453007, China

**Keywords:** demand prediction, intermittent time series, tensor decomposition, interval prediction, time series forecasting

## Abstract

Demand for spare parts, which is triggered by element failure, project schedule and reliability demand, etc., is a kind of sensing data to the aftermarket service of large manufacturing enterprises. Prediction of the demand for spare parts plays a crucial role in inventory management and lifecycle quality management for the aftermarket service of large-scale manufacturing enterprises. In real-life applications, however, demand for spare parts occurs randomly and fluctuates greatly, and the demand sequence shows obvious intermittent distribution characteristics. Additionally, due to factors such as reporting mistakes made by personnel or environmental changes, the actual data of the demand for spare parts are prone to abnormal variations. It is thus hard to capture the evolutional pattern of the demand for spare parts by traditional time series forecasting methods. The reliability of prediction results is also reduced. To address these concerns, this paper proposes a tensor optimization-based robust interval prediction method of intermittent time series for the aftersales demand for spare parts. First, using the advantages of tensor decomposition to effectively mine intrinsic information from raw data, a sequence-smoothing network based on tensor decomposition and a stacked autoencoder is proposed. Tucker decomposition is applied to the hidden features of the encoder, and the obtained core tensor is reconstructed through the decoder, thus allowing us to smooth outliers in the original demand sequence. An alternating optimization algorithm is further designed to find the optimal sequence feature representation and tensor decomposition factors for the extraction of the evolutionary trend of the intermittent series. Second, an adaptive interval prediction algorithm with a dynamic update mechanism is designed to obtain point prediction values and prediction intervals for the demand sequence, thereby improving the reliability of the forecast. The proposed method is validated using the actual aftersales data from a large engineering manufacturing enterprise in China. The experimental results demonstrate that, compared with typical time series prediction methods, the proposed method can effectively grab the evolutionary trend of various intermittent series and improve the accuracy of predictions made with small-sample intermittent series. Moreover, the proposed method provides a reliable elastic prediction interval when distortion occurs in the prediction results, offering a new solution for intelligent planning decisions related to spare parts in practical maintenance.

## 1. Introduction

In complicated equipment manufacturing enterprises such as shield tunneling, rail transportation, and wind energy, the cost of maintaining an inventory of spare parts generally accounts for more than 60% of inventory costs [1]. Qualified inventory optimization [2] and flexible scheduling of parts [3] are critical to improve the efficiency of aftermarket service in lifecycle product management [4]. Due to various uncertain factors, such as element failure, project schedule, safe inventory level, etc., there will be an inventory shortage of spare parts in warehouses which, in turn, triggers the demand for spare parts. The demand for spare parts can then serve as a kind of sensing data to monitor the maintenance efficiency and evaluate the aftermarket service quality. Accurate prediction of the demand for spare parts plays a supporting role in intelligent inventory optimization. However, in practical operations, parts planning is often linked to new online projects or associated with the unavailability of spare parts, resulting in sporadic demand for spare parts. The data distribution of the demand for spare parts therefore has intermittent characteristics. Precisely predicting the demand of intermittent time series is challenging in spare parts management for manufacturing enterprises.

Due to the intermittent characteristics of these demand data, predicting demand relies heavily on time series prediction. Currently, time series prediction methods can be divided into three categories [5]: (1) statistical methods (e.g., exponential smoothing [6] and moving average [7]); (2) machine learning methods (e.g., support vector regression (SVR) [8], random forests (RF) [9], and LightGBM (light gradient boosting machine) [10,11]); and (3) deep learning methods (e.g., recurrent neural networks (RNN) [12] and long short-term memory (LSTM) [13]). These methods are often applicable to time series with strong periodicity and apparent trends. However, there are some challenges involved in effectively extracting evolutionary patterns from time series with strong randomness, poor continuity, and, especially, small sample sizes, leading to low prediction accuracy for intermittent series. To solve this problem, Croston [14] improved the exponential smoothing algorithm by decomposing intermittent time series into zero-interval and demand sequences. The exponential smoothing algorithm was then applied to each sequence separately, and the results were weighted to improve prediction performance. Syntetos et al. [15] further improved the Croston algorithm and designed the Syntetos–Boylan approximation (SBA) method. The SBA method introduced a bias term (1−α/2) to mitigate the uncertainty of intermittent distributions. In addition, some studies proposed different metrics, such as the average demand interval (ADI) and the square of the coefficient of variation (CV2) [16], to explore intermittent characteristics to better extract intrinsic information from demand sequences [17]. Another typical approach is to perform hierarchical clustering on demand sequences [18], which divides the original sequences with weak overall patterns into multiple clusters with more significant patterns. Then, different regression algorithms can be applied to the clusters for prediction. Shi et al. [19] proposed the block Hankel tensor-autoregressive integrated moving average (BHT-ARIMA) model, which uses tensor decomposition [20] to extract the intrinsic correlations among multidimensional small-sample sequences. Although the aforementioned methods have achieved certain results, they still have some limitations. First, they are mostly based on the assumption that all sequence demands have predictive value and disregard the interference of abnormal values. In fact, in actual business, due to special events such as natural disasters, emergencies, market fluctuations, and other factors, some spare parts have abnormal demand patterns, which require manual analysis for recognition. Second, demand prediction results are random and unreliable. Operations still want to obtain trustworthy results regarding demand prediction, but the existing methods fail to make valid decisions when the prediction results are distorted.

There is also a similar concept, i.e., abnormal demand forecasting, which refers to the process of forecasting and analyzing abnormal demands that may occur in the future. Guo et al. [21] utilized the passenger flow characteristics extracted by SVR into LSTM to predict abnormal passenger flow. Liu et al. [22] combined statistical learning and linear regression to build a model of the relationship between price discounts and demand for medical consumables. Li et al. [23] proposed a multi-scale radial basis function (MSRBF) network to predict subway passenger flow under special events. Nguyen et al. [24] combined LSTM with SVM to detect outliers in a demand sequence, and then used an LSTM neural network to predict the occurrence of outliers. Li et al. [25] proposed a combination model of time series and regression analysis, plus dummy variables, to predict special factors related to passenger flow. These methods aim to predict the occurrence of abnormal but meaningful events in many fields. However, in the situation described by this paper, abnormal demands are commonly found due to reporting mistakes made by personnel or environmental changes. Such abnormal demands are harmful to the prediction of the intermittent time series, since the evolutionary pattern of the demand for spare parts is disturbed. There is no value in predicting abnormal demands if such demands have no predictability. This paper is, therefore, solely devoted to predicting normal demands against the disturbances induced by abnormal demands, instead of predicting the abnormal demands.

To solve the problems mentioned above, this paper employs a tensor decomposition technique to smooth the abnormal demands in demand sequences. Tensor Tucker decomposition decomposes a tensor into a set of factor matrices and one core tensor that can describe the intrinsic information from raw data. With the decomposed forms, tensor Tucker decomposition brings the potential to extract the evolutionary trend from intermittent time series. The concept of a prediction interval is further introduced to tackle the problem of high uncertainty and less reliability in the prediction results. The methodology is as follows: First, a tensor autoencoder network is constructed, which performs tensor Tucker decomposition on the output features extracted from the hidden layers of the autoencoder. Second, the core tensor is decoded and reconstructed with an alternating optimization strategy to obtain the optimal feature representation of intermittent time series. Third, an adaptive prediction interval (API) algorithm is developed with a dynamical update mechanism to obtain point prediction values and prediction intervals, thus improving the reliability of the prediction results. Finally, the performance of the proposed method is validated using a set of real-life aftersales data from a large engineering manufacturing enterprise from China.

The contributions of this paper can be summarized as follows:

(1) An intermittent series smoothing algorithm is proposed. By integrating tensor Tucker decomposition into a stacked autoencoder network with an alternately optimizing scheme, the proposed algorithm extracts the evolutionary trend of the intermittent time series under irregular noise interference. Compared with existing time series anomaly detection methods, the proposed algorithm does not require any pre-fixed detection thresholds, and can adaptively identify outliers in the series. It is highly suitable for smoothing the outliers in the intermittent time series. Moreover, the proposed algorithm is universally applicable, e.g., the stacked autoencoder can be easily replaced by other deep models;

(2) An adaptive prediction interval algorithm is designed. Different from the existing point prediction methods using a deterministic prediction model, this algorithm incorporates the prediction intervals with the point prediction, which can match the intermittent characteristics of demand sequence for spare parts. This algorithm provides an effective solution to address the uncertainty in the demand for spare parts. According to the literature survey, there is no related research about interval prediction, specifically for demand prediction.

## 2. Background

### 2.1. Multi-Way Delay Embedding Transform

The multi-way delay embedding transform (MDT) [26] is capable of embedding low-order data into a high-dimensional space, which can be used to construct Hankel matrices or block Hankel tensors. The tensor obtained by MDT possesses the characteristics of low rank, which can smooth the original data to make them easier to train. $v={\left(v_{1},\ldots ,v_{L}\right)}^{T}\in R^{L}$ denotes a vector that is transformed into a Hankel matrix with a delay of τ through MDT. This process is referred to Hankelization of the vector. The transform process is shown in Equation (1).

(1) $$H_{\tau }\left(vs.\right):=\left(\begin{array}{cccc} v_{1} & v_{2} & \cdots & v_{L-\tau +1}\\ v_{2} & v_{3} & \cdots & v_{L-\tau +2}\\ \vdots & \vdots & \ddots & \vdots \\ v_{\tau } & v_{\tau +1} & \cdots & v_{L} \end{array}\right)\in R^{\tau \times \left(L-\tau +1\right)}\frac{-b\pm \sqrt{b^{2}-4ac}}{2a}$$

First, the duplication matrix $S\in {\left\{0,1\right\}}^{\tau \times \left(L-\tau +1\right)\times L}$ is constructed with a delay of τ, as shown in Equation (2).

(2) $$S^{T}={\left(\begin{array}{cccc} I_{\tau } & & & \\ {}_{} & I_{\tau } & & \\ & & \ddots & \\ & & & I_{\tau } \end{array}\right)}_{\tau \times \tau }T$$

The vector *v* is transformed into a Hankel matrix denoted by $H_{\tau }\left(vs.\right)$. The duplication matrix *S* is essentially a linear transformation. The vectorization expansion is shown in Equation (3):

(3) $$vec\left(H_{\tau }\left(vs.\right)\right)=Sv,\;Sv\in R^{\tau \times \left(L-\tau +1\right)}$$

where vec(·) expands the matrix along the column direction. The Hankel matrix through delay embedding can be shown in Equation (4).

(4) $$\begin{array}{c} H_{\tau }\left(vs.\right)=fold_{\left(L,\tau \right)}\left(Sv\right):=v_{H},\\ fold_{\left(L,\tau \right)}:R^{\tau \times \left(L-\tau +1\right)}\to R^{\tau \times \left(L-\tau +1\right)} \end{array}$$

where $fold_{\left(L,\tau \right)}$ folds a vector into a matrix.

The inverse transform of multi-way delay embedding for vectors can convert the data from a high-dimensional space to a lower-dimensional target space. This can be calculated using Equations (5) and (6).

(5) $$H_{\tau }^{-1}\left(V_{H}\right)=S^{†}vec\left(V_{H}\right)$$

(6) $$S^{†}:={\left(S^{T}S\right)}^{-1}S^{T}$$

where † is the Moore–Penrose inverse matrix [27].

### 2.2. Tensor Decomposition

Tensor decomposition aims to decompose high-order tensor data into low-rank matrices or vectors [28]. It is commonly applied in data compression, dimensionality reduction, feature extraction, etc. Tensor Tucker decomposition decomposes an *N*th-order tensor $\chi \in R^{I_{1}\times I_{2}\times \cdots \times I_{N}}$ into the product of a core tensor $\varsigma_{t}\in R^{J_{1}\times J_{2}\times \cdots \times J_{N}}$ and *N* factor matrices $U^{\left(n\right)}\in R^{I_{n}\times J_{n}}$, as shown in Equation (7). The factor matrices obtained from Tucker decomposition represent the principal components of the tensor’s modular expansion, while the core tensor captures the correlations between these components.

(7) $$\chi =\varsigma \times {}_{1}U^{\left(1\right)}\times {}_{2}U^{\left(2\right)}\cdots \times {}_{N}U^{\left(N\right)}$$

where $\varsigma \times {}_{1}U^{\left(n\right)}$ is the *n*-mode product of the modular (*n*) expansion of tensor *S* and matrix $U^{\left(n\right)}\in R^{I_{n}\times J_{n}}$:

(8) $$\begin{array}{c} {\left[\varsigma \times U^{\left(n\right)}\right]}_{j_{1}\cdots j_{n-1}i_{n}j_{n+1}\cdots j_{N}}=\sum_{j_{n}=1}^{J_{n}}g_{j_{1}\cdots j_{n-1}i_{n}j_{n+1}\cdots j_{N}}u_{i_{n}j_{n}}\\ \varsigma \times U^{\left(n\right)}\in R^{J_{1}\times J_{2}\times \cdots \times J_{N}} \end{array}$$

With the above equation, one data point in the tensor can be expanded by Equation (9) as:

(9) $$x_{i_{1}i_{2}\cdots i_{N}}=\sum_{j_{1},j_{2},\cdots ,j_{N}}^{}g_{j_{1}\cdots j_{N}}u_{i_{1}j_{1}}^{\left(1\right)}u_{i_{2}j_{2}}^{\left(2\right)}\cdots u_{i_{3}j_{3}}^{\left(3\right)}$$

**Figure 1:**
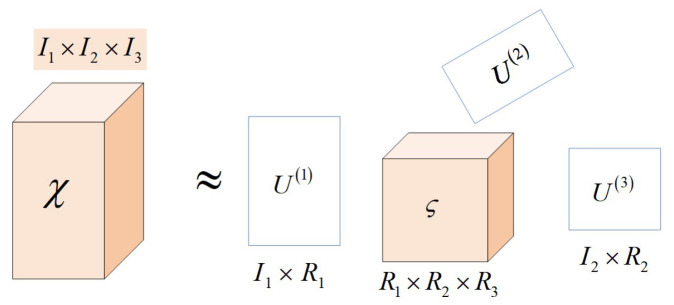
Illustration of three-order tensor Tucker decomposition.

Figure 1 illustrates a three-order tensor decomposed using Tucker decomposition, resulting in the product of a smaller core tensor and three factor matrices.

### 2.3. LightGBM

LightGBM, developed by Microsoft engineers [10], is a gradient-boosting framework based on decision trees. By utilizing feature parallelism during its training process, LightGBM assigns discrete features to multiple bins and constructs decision trees using histogram algorithms, which provides a quick and efficient training mechanism for LightGBM. Additionally, LightGBM employs a sparse feature algorithm to significantly reduce memory consumption, which is suitable for training with extremely large-scale data. Moreover, LightGBM utilizes a leaf-wise tree growth strategy, which leads to faster convergence and higher accuracy compared to traditional level-wise strategies.

## 3. Proposed Method

In this section, a tensor optimization-based robust interval prediction method of intermittent time series is presented. This method consists of two parts: (1) a sequence smoothing network based on tensor decomposition and a stacked autoencoder, which aims to smooth anomalous demand values in the original sequence and to extract the evolutionary trend of demand as well; and (2) an adaptive prediction interval algorithm, which aims to construct a reliable prediction interval to avoid the oversupply risk or inventory shortage caused by inaccurate predictions. In this method, the role of tensor Tucker decomposition is: (1) extracting core tensors from the demand sequence for representing the evolutionary trend; and (2) smoothing the outliers in the sequence. The negative interference of anomalous demands can then be effectively reduced under an unsupervised mode. Moreover, training a LightGBM model with core tensors can better mine the trend information from the demand sequence. Tensor Tucker decomposition is believed suitable for intermittent time series forecasting with small samples.

**Figure 2:**
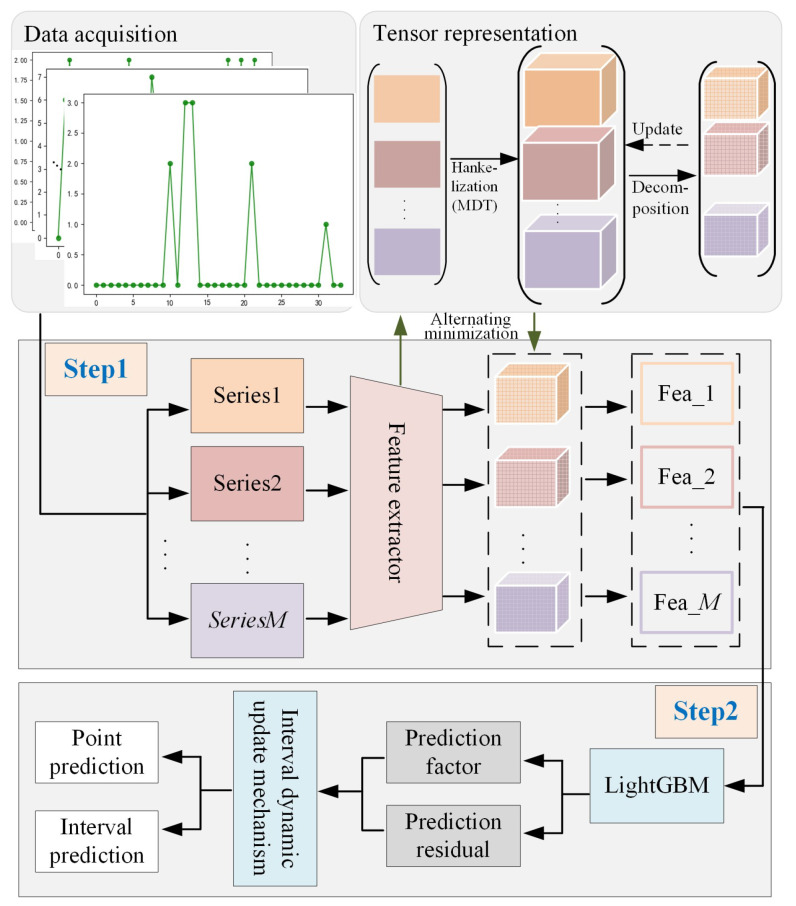
Flowchart of the proposed method.

The flowchart of the proposed method is shown in Figure 2. First, the hidden features are extracted from the original data using a stacked autoencoder, then tensor Tucker decomposition is performed on the hidden features to obtain the core tensors. An alternating optimization scheme is designed to obtain the optimal core tensors by alternately updating the autoencoder parameters and tensor factor matrices. Second, an adaptive interval prediction algorithm is constructed. The interval is calculated using the predicted values and prediction residuals from LightGBM estimators. Finally, a dynamic update mechanism is used to adjust the width of prediction interval. The detailed implementation will be presented as follows.

### 3.1. Sequence Smoothing Network

Denote the demand sequence by $X=\left\{x_{1},x_{2},\ldots ,x_{m}\right\}\in R^{m\times n}$, where *m* indicates the sample number and *n* represents the time dimension. The sequence can be encoded and mapped into a hidden layer, as shown in Equation (10).

(10) h=f(Wx+b)

where *h* represents the hidden layer features, f(·) is the activation function of the encoding layer, and $W\in R^{r}$ and $b\in R^{r}$ are the weight matrix and bias vector of the encoding layer, respectively. The obtained feature set is denoted by $H=\left\{h_{1}^{},h_{2}^{},\cdots ,h_{m}^{}\right\}\in R^{m\times l}$, where *l* represents the dimension of the deep features.

In order to adequately represent the temporal information between samples in the feature set *H*, an MDT with the operations of multi-linear duplication and multi-way folding is employed to transform the original sequence to a three-order tensor. This process can also reduce noise disturbance. Denote by $H=\left\{h_{1}^{},h_{2}^{},\cdots ,h_{m}^{}\right\}\in R^{m\times l}$ the tensor of $X=\left\{X_{1},\ldots ,X_{m-\tau +1}\right\}\subseteq R^{l\times \tau \times (m-\tau +1)}$, then the MDT for X can be defined as:

(11) $$X_{i}=H_{\tau }\left(H\right)=Fold_{\left(m,\tau \right)}\left(H\times {}_{1}S_{1}\times \cdots \times {}_{m-\tau +1}S_{m-\tau +1}\right)$$

where τ and m represent the time window size and sample length, respectively, and *S* is a duplication matrix. In this paper, τ is set to 6.

With the tensors constructed by MDT, the Tucker decomposition technique is introduced to obtain the three-order core tensor $G=\left\{g_{1}^{1},\ldots ,g_{{}_{m-\tau +1}}^{l}\right\}\in R^{l\times \tau \times (m-\tau +1)}$ that represents the essential information of the sequence:

(12) $$\begin{array}{c} G=X\times {}_{1}{U^{\left(1\right)}}^{T}\times {}_{2}{U^{\left(2\right)}}^{T}\times \cdots \times {}_{V}{U^{\left(v\right)}}^{T}\\ s.t.\;{U^{\left(v\right)}}^{T}U^{\left(v\right)}=I,v=1,\cdots ,V \end{array}$$

where ${\left\{U^{\left(v\right)}\right\}}_{v=1}^{V}$ is the projection matrix and can maximally preserve the temporal continuity between core tensors, where the superscript *v* represents the tensor dimension, and m−τ+1 represents the sample length after reconstruction. Equation (12) means that the decomposition result consists of a core tensor and a series of factor matrices. Obviously, the core tensor G contains the intrinsic information of an evolutionary trend. By optimizing ${\left\{U^{\left(v\right)}\right\}}_{v=1}^{V}$, the inherent correlation between feature sequences can be sufficiently captured, while noise interference can also be reduced. The loss of tensor reconstruction can be calculated as:

(13) $$\begin{array}{c} L_{Tensor}=\min{∥X-\hat{X}∥}_{F}^{2} \end{array}$$

where X is the original tensor, and $X=G\times {}_{1}U^{\left(1\right)}\times {}_{2}U^{\left(2\right)}\times \cdots \times {}_{H}U^{\left(H\right)}$ is the tensor reconstructed from the factor matrix ${\left\{U^{\left(v\right)}\right\}}_{v=1}^{V}$.

To implement the decoding operation, it is necessary to transform the core tensor G back to the original input space. Here, the inverse MDT transform [6] is applied to G by reversing the transformation along the time dimension to obtain a second-order core tensor $G^{\prime }=\left\{g_{1}^{\prime },\ldots ,g_{m}^{\prime }\right\}\in R^{m\times l}$, as shown in Equation (14). The core tensor $G^{\prime }$ is then used as the input of decoding to update the network.

(14) $$G^{\prime }=H\left(G\right)=Unfold_{\left(m,\tau \right)}\left(G\right)\times {}_{1}{S_{1}}^{†}\times \cdots \times {}_{m-\tau +1}S_{m-\tau +1}^{†}$$

where † is the Moore–Penrose pseudo-inverse.

Through decoding $G^{\prime }$, the reconstructed data $\hat{x}$ can be obtained as follows:

(15) $$\hat{x}=f^{*}\left(W^{*}g^{\prime }+b^{*}\right)$$

where $f^{*}\left(\cdot \right)$ is the activation function of the decoding layer, $W^{*}\in R^{n\times r}$ is the weight matrix of the decoding layer, and $b^{*}\in R^{n}$ is the bias vector of the decoding layer. Consequently, the reconstruction loss of the autoencoder can be calculated as:

(16) $$\underset{}{L_{AE}=}\frac{1}{m}\sum_{i=1}^{m}\frac{1}{2}{∥\hat{x}-x∥}^{2}+\frac{\lambda }{2}\left({{∥W∥}_{F}}^{2}+{{∥W^{*}∥}_{F}}^{2}\right)$$

where λ is the weight decay parameter and $∥\cdot ∥$ is the Frobenius norm.

Based on the aforementioned analysis, the whole loss function is:

(17) $$L_{loss}=\sum_{i=1}^{M}L_{AE}+\eta L_{Tensor}$$

Minimizing Equation (17) can smooth anomalous demands in the demand sequence and extract the evolutionary trend of the demand for spare parts. The key idea of this process is to reduce the significant deviations of anomalous demands, making them suitable for intermittent sequence anomaly detection. The crucial aspect of this process lies in utilizing the core tensor to represent the evolutionary trend. As Equation (12) indicates, ${\left\{U^{\left(v\right)}\right\}}_{v=1}^{V}$ is randomly initialized. Therefore, the optimization of tensor decomposition in minimizing Equation (17) is required to obtain the optimal representation of the core tensors.

### 3.2. Alternating Optimization Scheme

Minimization of Equation (17) includes the optimization of $L_{AE}$ and $L_{Tensor}$. The $L_{AE}$ can be solved using a stochastic gradient descent (SGD) strategy [29]. However, SGD cannot be directly applied to tensor decomposition. Therefore, this paper adopts an alternating optimization strategy: fix ${\left\{U^{\left(v\right)}\right\}}_{v=1}^{V}$, and update $W,W^{*}$; then fix the updated $W,W^{*}$, and update ${\left\{U^{\left(v\right)}\right\}}_{v=1}^{V}$. These two steps are performed alternately until convergence. It should be noted that, since the number of tensor optimization iterations is typically smaller than the number of $W,W^{*}$ updates, updating ${\left\{U^{\left(v\right)}\right\}}_{v=1}^{V}$ is set to stop when the difference between two consecutive tensor optimizations of ${\left\{U^{\left(v\right)}\right\}}_{v=1}^{V}$ is less than a specific threshold. $W,W^{*}$ is updated until the model converges. With the initialized *W* and $W^{*}$, the specific optimization process is as follows:

(1) Fix ${\left\{U^{\left(v\right)}\right\}}_{v=1}^{V}$, and update $W,W^{*}$;

1. Encoding stage: the partial derivatives of $L_{AE}$ with respect to the parameters are:

(18) $$\left\{\begin{array}{c} \frac{\partial L_{AE}}{\partial W}=\frac{2}{M}\sum_{m=1}^{M}\left({\hat{x}}^{\left(m\right)}-x^{\left(m\right)}\right)\cdot \frac{\partial {\left({\hat{x}}^{\left(m\right)}-x^{\left(m\right)}\right)}^{T}}{\partial W}+2\lambda \frac{\partial ({{∥W∥}_{F}}^{2}+{{∥W^{*}∥}_{F}}^{2})}{\partial W}\\ \frac{\partial L_{AE}}{\partial b}=\frac{2}{M}\sum_{m=1}^{M}\left({\hat{x}}^{\left(m\right)}-x^{\left(m\right)}\right)\cdot \frac{\partial {\left({\hat{x}}^{\left(m\right)}-x^{\left(m\right)}\right)}^{T}}{\partial b} \end{array}\right.$$

The partial derivatives corresponding to the error term on each sample can be obtained by:

(19) $$\left\{\begin{array}{c} \frac{\partial ({\hat{x}}^{\left(m\right)}-x^{\left(m\right)})}{\partial W}=\sum_{m=1}^{M}\frac{\partial {\left({\hat{x}}^{\left(m\right)}\right)}^{T}}{\partial W}=f^{\prime }\cdot diag\left(X^{\left(m\right)}\right)\in R^{1\times r}\\ \frac{\partial ({\hat{x}}^{\left(m\right)}-x^{\left(m\right)})}{\partial b}=\sum_{m=1}^{M}\frac{\partial {\left({\hat{x}}^{\left(m\right)}\right)}^{T}}{\partial b}=f^{\prime }\cdot 1_{r}\in R^{1\times 1} \end{array}\right.$$

where “·” represents the dot product operator, diag(·) is the diagonal matrix, $1_{r}$ is the unit column vector of size *r*, and $f^{\prime }$ is the derivative of the activation function;

2. Decoding stage: the partial derivatives of $L_{AE}$ with respect to the parameters are:

(20) $$\left\{\begin{array}{c} \frac{\partial L_{AE}}{\partial W^{*}}=\frac{2}{M}\sum_{m=1}^{M}\left({\hat{x}}^{\left(m\right)}-x^{\left(m\right)}\right)\cdot \frac{\partial {\left({\hat{x}}^{\left(m\right)}-x^{\left(m\right)}\right)}^{T}}{\partial W^{*}}+2\lambda \frac{\partial ({{∥W∥}_{F}}^{2}+{{∥W^{*}∥}_{F}}^{2})}{\partial W^{*}}\\ \frac{\partial L_{AE}}{\partial b^{*}}=\frac{2}{M}\sum_{m=1}^{M}\left({\hat{x}}^{\left(m\right)}-x^{\left(m\right)}\right)\cdot \frac{\partial {\left({\hat{x}}^{\left(m\right)}-x^{\left(m\right)}\right)}^{T}}{\partial b^{*}} \end{array}\right.$$

For easy analysis, the error propagation term (i.e., the derivative of the error term with respect to the hidden layer output) is briefly denoted by:

(21) $$\delta_{H^{\left(m\right)}}=\left(\frac{\partial ({\hat{x}}^{\left(m\right)}-x^{\left(m\right)})}{\partial H^{\left(m\right)}}\right)$$

Furthermore, following the chain rule, we have:

(22) $$\left\{\begin{array}{c} \frac{\partial ({\hat{x}}^{\left(m\right)}-x^{\left(m\right)})}{\partial W^{*}}=\delta_{H^{\left(m\right)}}\cdot \frac{\partial H^{\left(m\right)}}{\partial W^{*}}=\delta_{H^{\left(m\right)}}\cdot {\left(f^{\prime }⊙diag\left(H^{\left(m\right)}\right)\right)}^{T}\\ \frac{\partial ({\hat{x}}^{\left(m\right)}-x^{\left(m\right)})}{\partial b^{*}}=\delta_{H^{\left(m\right)}}\cdot \frac{\partial H^{\left(m\right)}}{\partial b^{*}}=\delta_{H^{\left(m\right)}}\cdot {\left(f^{\prime }⊙1_{r}\right)}^{T} \end{array}\right.$$

where “·” and diag(·) have the same meaning as in the encoding stage;

3. Substitute Equations (19) and (22) into Equations (18) and (20), respectively, and the model parameters can be updated as:

(23) $$\left\{\begin{array}{c} W^{\left(l+1\right)}=W^{\left(l\right)}-\xi \cdot \frac{\partial L_{AE}}{\partial W}\left|{}_{W=W^{\left(l\right)}}\right.\\ b^{\left(l+1\right)}=b^{\left(l\right)}-\xi \cdot \frac{\partial L_{AE}}{\partial b}\left|{}_{b=b^{\left(l\right)}}\right.\\ {W^{*}}^{\left(l+1\right)}={W^{*}}^{\left(l\right)}-\xi \cdot \frac{\partial L_{AE}}{\partial W^{*}}\left|{}_{W^{*}={W^{*}}^{\left(l\right)}}\right.\\ {b^{*}}^{\left(l+1\right)}={b^{*}}^{\left(l\right)}-\xi \cdot \frac{\partial L_{AE}}{\partial b^{*}}\left|{}_{b^{*}={b^{*}}^{\left(l\right)}}\right. \end{array}\right.$$

where ξ is the learning rate.

(2) Fix $W,W^{*}$ and update ${\left\{U^{\left(v\right)}\right\}}_{v=1}^{V}$.

Updating ${\left\{U^{\left(v\right)}\right\}}_{v=1}^{V}$ can be achieved by minimizing the tensor reconstruction error as shown in Equation (13). Since ${\left\{U^{\left(v\right)}\right\}}_{v=1}^{V}$ is an orthogonal matrix, Equation (13) can be rewritten as follows:

(24) $$\begin{array}{c} {∥X-\hat{X}∥}_{F}^{2}\;=\;{∥vec\left(X\right)-\left(U^{\left(V\right)}⊗U^{\left(V-1\right)}⊗\cdots ⊗U^{\left(1\right)}\right)\cdot vec\left(G\right)∥}_{F}^{2}\\ \;\;\;\;\;\;\;\;\;\;\;\;={∥vec\left(X\right)-\left(U^{\left(V\right)}⊗U^{\left(V-1\right)}⊗\cdots ⊗U^{\left(1\right)}\right)\cdot {\left(U^{\left(V\right)}⊗U^{\left(V-1\right)}⊗\cdots ⊗U^{\left(1\right)}\right)}^{T}\cdot vec\left(X\right)∥}_{F}^{2}\\ \;\;\;\;\;\;\;\;\;\;\;\;={∥vec\left(X\right)∥}_{F}^{2}-{∥U^{T}\cdot vec\left(X\right)∥}_{F}^{2} \end{array}$$

where $U^{T}=U^{\left(H\right)}⊗U^{\left(H-1\right)}⊗\cdots ⊗U^{\left(1\right)}$. To minimize Equation (24), we should maximize the following equation:

(25) $$\begin{array}{c} \begin{array}{c} {∥U^{T}\cdot vec\left(X\right)∥}_{F}^{2}\;=\;\left\{\begin{array}{c} {∥U^{{\left(1\right)}^{T}}\cdot A^{\left(1\right)}\cdot \left(U^{\left(H\right)}⊗U^{\left(H-1\right)}⊗\cdots ⊗U^{\left(2\right)}\right)∥}_{F}^{2}\\ {∥U^{{\left(2\right)}^{T}}\cdot A^{\left(2\right)}\cdot \left(U^{\left(H\right)}⊗U^{\left(H-1\right)}⊗\cdots ⊗U^{\left(1\right)}\right)∥}_{F}^{2}\\ \;\;\;\;\;\;\;\;\;\;\;\;\;\;\;\;\;\;\;\;\;\;\;\;\;\;\;\cdots \\ {∥U^{{\left(H\right)}^{T}}\cdot A^{\left(H\right)}\cdot \left(U^{\left(H-1\right)}⊗U^{\left(H-2\right)}⊗\cdots ⊗U^{\left(1\right)}\right)∥}_{F}^{2} \end{array}\right. \end{array} \end{array}$$

where $A^{\left(i\right)}$ is the unfolding matrix of the tensor X along the *i*-th dimension. The alternating least squares method [30] can be used to solve it, where each factor matrix in different directions is fixed sequentially to find the least squares solution. This process is shown in Equation (26):

(26) $$U_{k+1}^{\left(v\right)}=\underset{{\left\{U^{\left(v\right)}\right\}}_{v=1}^{V}}{\arg\min}{∥\hat{X}-G\times {}_{M}U_{k}^{{}^{\left(V\right)}}\times {}_{M-1}\cdots \times {}_{1}U_{k}^{{}^{\left(1\right)}}∥}_{F}^{2}$$

### 3.3. Adaptive Prediction Intervals (API)

After obtaining the optimal feature representation, this paper designs the API algorithm to generate the point prediction values and reliable prediction intervals. The details of the API algorithm are presented as follows. The API algorithm consists of two stages: training and prediction. At the training stage, a fixed number of LightGBM estimators are fitted from a subset of training data. Then, the predicted value from all the LightGBM estimators is aggregated using the leave-one-out (LOO) strategy to generate LOO prediction factors and residuals. At the prediction stage, the API algorithm calculates the LOO predicted value by averaging the prediction factors from each test sample. With the predicted values, the center of the prediction interval is determined. The prediction intervals are then established using the LOO residuals. The width of prediction interval is updated using a dynamic updating strategy. The specific implementation steps are as follows:

First, a LightGBM model *f* is trained using the training samples ${\left\{\left(x_{t},y_{t}\right)\right\}}_{t=1}^{T}$, and the prediction interval at the *t*-th time step is calculated as:

(27) $${\hat{C}}_{t}^{\alpha }=\left[{\hat{f}}_{-t}\left(x_{t}\right)+q_{\hat{\beta }},{\hat{f}}_{-t}\left(x_{t}\right)+q_{\left(1-\alpha +\hat{\beta }\right)}\right]$$

where α is the significance factor, ${\hat{f}}_{-t}$ represents the *t*-th estimator of *f*, $q_{\hat{\beta }}$ is the $\hat{\beta }$-quantile on ${\left\{{\hat{\varsigma }}_{i}\right\}}_{i=t-1}^{t-T}$, and $q_{\left(1-\alpha +\hat{\beta }\right)}$ is the $\left(1-\alpha +\hat{\beta }\right)$-quantile on ${\left\{{\hat{\varsigma }}_{i}\right\}}_{i=t-1}^{t-T}$. The LOO prediction residual ${\hat{\varsigma }}_{i}$ and $\hat{\beta }$ are defined as follows:

(28) $${\hat{\varsigma }}_{i}=y_{i}-{\hat{f}}_{-t}\left(x_{t}\right)$$

(29) $$\hat{\beta }=\underset{\beta \in [0,\alpha ]}{\arg\min}\left({\hat{f}}_{-t}\left(x_{t}\right)+q_{\left(1-\alpha +\hat{\beta }\right)}-{\hat{f}}_{-t}\left(x_{t}\right)+q_{\hat{\beta }}\right)$$

Second, the interval center is ${\hat{f}}_{-t}\left(x_{t}\right)$, and the interval width is the difference between the $\left(1-\alpha +\hat{\beta }\right)$ and $\hat{\beta }$-quantiles on the past *T* residuals.

However, the obtained interval values cannot adequately address the problem of large fluctuations of demands for different spare parts. This section proposes an adaptive update mechanism to improve the reliability of the prediction interval. The specific step is as follows: First, each demand sequence is divided into a zero-interval sequence (i.e., the gap between two subsequent demands occur) and a non-zero sequence (i.e., the real demand values). The squared coefficient of variation is then calculated for the two sequences, which are denoted by $cv_{1}$ and $cv_{2}$, respectively. A larger coefficient indicates greater sequence fluctuation, thus requiring a larger interval width for prediction, and vice versa. Second, initialize the interval width parameter α=0.1 and update α by:

(30) $$\alpha =\alpha +cv_{1}+cv_{2}$$

The interval update mechanism, as shown in Equation (30), can improve the rationality and reliability of the prediction interval for demand sequences with different volatility and intermittency characteristics. Obviously, different values of interval widths will directly affect the decision of inventory management, e.g., spare part coverage rate. The spare part coverage rate, reflected by the interval coverage rate in this paper, is defined as the ratio of the number of demands covered by the interval to the total number of demands in the sequence. The mechanism begins by setting an initial interval with a larger width, and then reduces the width to meet the fluctuations in the demand sequence. The update process is stopped when the interval width reaches a certain threshold. In this study, the threshold of the interval width is determined just by the interval coverage rate. The setting of the coverage rate relies heavily on business logic. Different kinds of maintenance tasks or enterprises have different requirement for the setting. In this experiment, we received help from the maintenance engineers from our cooperative enterprise and set the lower limit of coverage rate to be 60%. We also observe that, with this threshold, the prediction results become stable, which indicates that the threshold runs well.

## 4. Validation Results and Analysis

### 4.1. Experimental Settings

**Figure 3:**
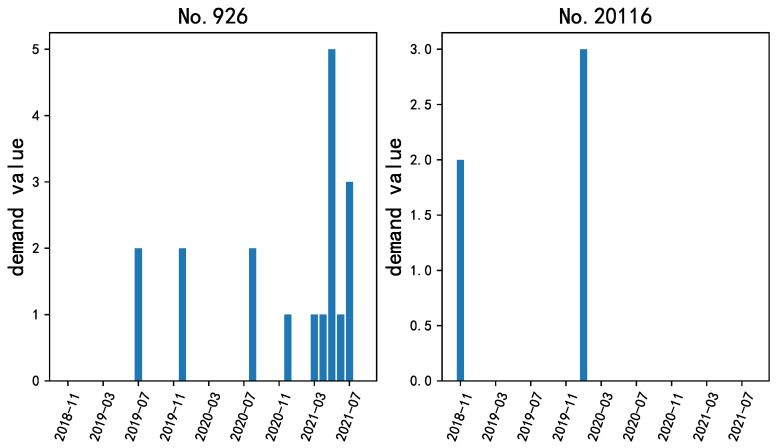
Example of demands for different spare parts used in this experiment. Due to commercial confidentiality requirements, only the part index, instead of its specific name, is given here.

The experimental data for validation are the real-life demand data of spare parts from a large engineering manufacturing enterprise from China. The data set contains 75 sequences of different spare parts, in which each sequence covers 34 months from November 2018 to August 2021. The training data are the demand values in the first 33 months, while the data of the last month are for the test. The enterprise usually has one month in advance to make inventory plans and implement the allocation. Therefore, in this experiment we mainly focus on the prediction value of the last month in the whole sequence. To visualize the intermittent characteristic of the demand data, we randomly select two spare parts and illustrate their demand sequences in Figure 3.

For a fair comparison, we introduce six representative methods of time series forecasting in this experiment. These six methods are applicable for different kinds of time series. It is worth noting that BHT-ARIMA, which also adopts tensor decomposition and joint optimization strategy, can be viewed as the SOTA method for intermittent time series forecasting. See Table 1 for detailed information.

In the experiment, the parameters α and β in the Croston method are set in the range of [0.13, 0.26], and the step size is set to 0.07. The parameters *p* and *q* of BHT-ARIMA are set to 1 and 3, respectively. For ARIMA, the parameters *p* and *q* are set to 2 and 3, respectively. For Random Forest, a grid search strategy is adopted to select the optimal parameters, with the parameters determined as max_depth = 5, n_estimators = 30, learning = 0.05, and num_leaves = 20. The LSTM hidden layer is set to 20, and the learning rate is 0.001.

### 4.2. Result Analysis

**Figure 4:**
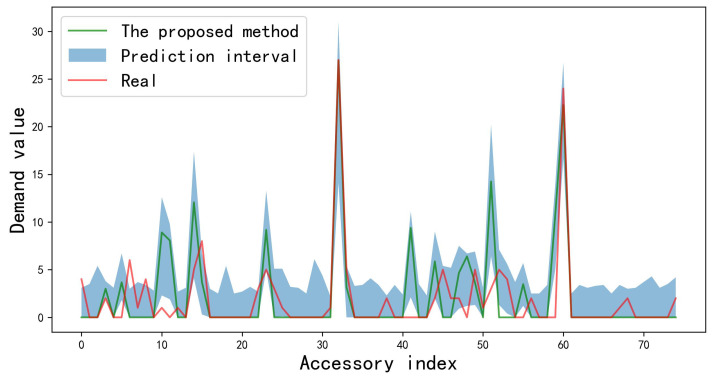
Prediction results of the proposed method on a total of 75 demand sequences.

In this experiment, the demand data of the first 33 months are used as training and the demand data of the 34th month is for the test. The predicted values and prediction intervals obtained by the proposed method are shown in Figure 4.

As suggested by the enterprise’s engineers, a prediction result within a range of ±30% around the real values can be regarded correct. From Figure 4, our method works well when dealing with high-demand data. The reason is that, besides the unsupervised feature extraction capability of the autoencoder network, the core tensors can represent the evolutionary trend of the raw demand data well. More importantly, when the prediction results are heavily biased, the prediction intervals obtained by the proposed method can effectively cover the true value. It is clear that the prediction results by the proposed method can improve the decision-making ability of enterprises in inventory management.

**Figure 5:**
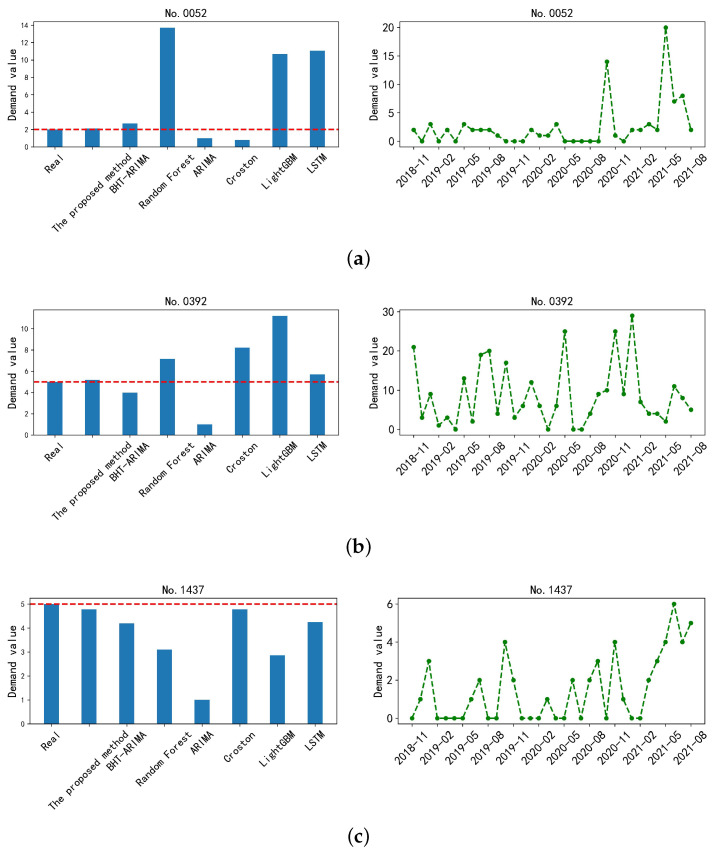
Comparative results of different prediction methods on the demand sequences for the spare parts, respectively, indexed by (**a**) No. 0052, (**b**) No. 0392, and (**c**) No. 1437. The demands for the three spare parts are randomly selected for validation and appear different levels of intermittent distribution characteristic. The left column is the prediction results, while the right column is the raw sequence for reference.

Figure 5 shows the comparison of different prediction methods on the demand sequences with different degrees of intermittent distribution.

From Figure 5, the zero-interval of the No.1437 sequence is relatively stable, so the Croston method can achieve good results. However, the Croston method performs worse than the other methods on the sequences No.0392 and No.0052 with large demand fluctuations. It indicates that Croston is only suitable for the intermittent series with a stationary zero-interval. The other conventional methods also have similar effects. These methods all have certain limitations and are only applicable to certain types of intermittent time series. Our method outperforms the other methods. The key part of our method is the introduction of tensor decomposition for smoothing anomalous demands, which is beneficial for the effective extraction of evolutionary trends from intermittent time series. With no surprise, our method shows superiority in the forecasting of intermittent time series with different distribution characteristics.

**Figure 6:**
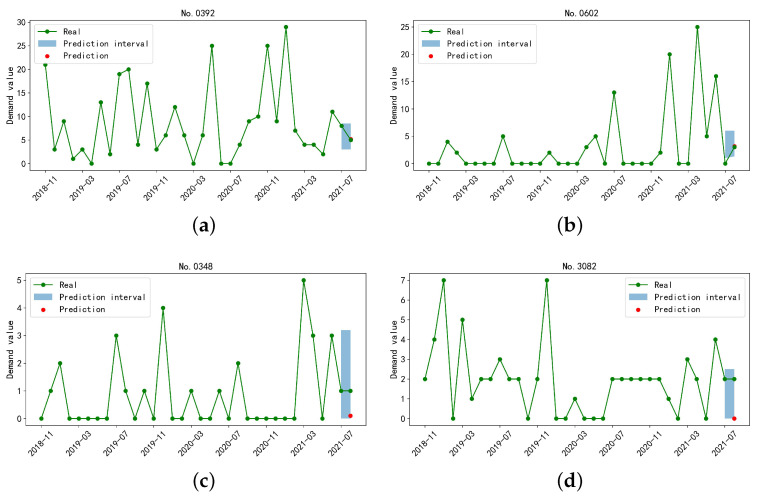
Prediction results of four parts sequences (**a**–**d**) with different intermittent distribution characteristics.

We further evaluate the effectiveness of the designed prediction interval. As stated above, a reliable prediction interval is able to provide a reference for inventory management, even if the point prediction is heavily biased or distorted. Figure 6 shows the prediction interval and predicted value of the demand sequences No. 0392, 0602, 0348, and 3082.

From Figure 6, the point prediction values of the sequences No. 0392 and No. 0602 are more accurate than the others. The prediction intervals also cover the real values, while the interval range remains small. On the contrary, the demand number in the sequences No. 0348 and No. 3082 is smaller, while the deviation of point prediction results is rather large. The prediction interval can cover the real value, so the enterprise can obtain precise information for the spare parts in this interval and make a more reliable plan on inventory management.

For numerical comparison, we introduce three commonly-used error metrics: MAE, RMSE, and RMSSE. The prediction errors obtained by different methods are listed in Table 2. Our method obtains the lowest prediction error in terms of all three metrics. With these results, our method is believed to provide a new reliable solution for enterprises to realize inventory management and parts scheduling.

The mainstream method in this field is the demand prediction method based on deep learning models. One advantage of the proposed method, compared to the current mainstream deep learning model, is introduction of tensor decomposition. By applying tensor decomposition, the core tensor can be extracted from the demand sequences. This effectively mitigates the impact of outliers in the demand sequences and diminishes their interference on the prediction results. Additionally, by utilizing LightGBM prediction with the core tensor, the proposed method can better capture the demand trend information in the demand sequences. The proposed method is then more suitable than deep learning methods for predicting intermittent time series with small samples.

We must point out the potential disadvantages of tensor Tucker decomposition. Actually, the process of tensor decomposition is rather computationally expensive. In the experiment, each round of tensor decomposition needs about 0.6 s, while the alternating optimization needs 3.07 s on average. The total cost of the proposed method is 30 s per round. In contrast, LSTM takes an average of 2.15 s per round, and the other shallow models like Croston, Random Forest, and ARIMA needs much less time. The high cost raises the potential risks of the proposed method for applications that require high real-time performance.

## 5. Conclusions

In this paper, a new tensor optimization-based robust interval prediction method of intermittent time series is proposed to forecast the demand for spare parts. With the introduction of tensor decomposition, the proposed method can smooth the anomalous demand while preserving the intrinsic information of evolutionary trend from the demand sequences. Moreover, to tackle the distorted or biased prediction results of intermittent time series, the API algorithm is designed to transform traditional point prediction to adaptive interval prediction. The proposed method is able to provide a trustworthy interval for the prediction results, and can accurately reflect the uncertainty of the prediction results.

This study is fundamental to develop the effect of inventory management. As a typical extended application, the proposed method is critical to update the current safety stock model to a dynamic version by integrating the evolutionary trend of demand for spare parts. For instance, the prediction results from this paper can adjust the upper bound of safety stock to match the fluctuations in the actual demands. We believe this operation can decrease the cost level while keeping maintenance.

Another interesting issue is the correlation among the demand sequences for different spare parts. The demand for two spare parts is probably correlated due to many factors such as project cycle, climatic influence, failure probability, etc. As proven by many prior works, the correlation information between two time series is valuable. Especially for intermittent time series prediction, analyzing the correlation among some sequences is believed to improve the predictability, which is critical for intermittent time series. The joint prediction of two or more sequences is able to enhance the prediction performance in terms of accuracy and numerical stability. We plan to explore the utilization of correlation information in the future work. A feasible implementation is running adaptive clustering algorithms with profile coefficients to determine the proper sequence clusters before the prediction, which is expected to improve the predictability. We can further adopt multi-output regression or multi-task learning algorithms to achieve joint prediction on the sequences in the same cluster. We plan to explore the utilization of correlation information in the future work.

In our future work, transfer learning will be studied for the prediction of demand for spare parts. In actual enterprises, the demand data are usually insufficient, especially for newly deployed equipment. Considering the distinction between different types of equipment, we plan to build a transfer learning model to incorporate the evolutionary information of the demand from available equipment. The reliability of the prediction interval is also an interesting work. It is necessary to find a reliable method to converge the prediction interval into a prediction point with a high confidence level.

## Figures and Tables

**Table 1:** Six prediction methods for comparison.

Method Name	Type	Implementation
Croston [14]	Intermittent time series forecasting	Exponential smoothing
BHT-ARIMA [19]		Joint optimization with ARIMA and tensor decomposition
ARIMA [31]	One-dimensional time series forecasting	Autoregressive modeling with moving average
Random Forest [9]	Multidimensional time series forecasting	Ensemble prediction
LightGBM [10]		Decision trees using histogram algorithm
LSTM [13]	Temporal deep learning method	Modeling long-term dependencies with memory units

**Table 2:** Numerical comparison of different methods in terms of three error metrics on all 75 demand sequences.

Algorithm	MAE	RMSE	RMSSE
Random Forest	1.85	2.77	0.79
ARIMA	1.99	4.40	0.73
Croston	1.77	2.79	0.84
LightGBM	1.85	2.80	0.78
LSTM	1.74	3.20	0.91
BHT-ARIMA	1.67	2.73	0.71
Our method	1.64	2.57	0.58

## Data Availability

Not applicable.
